# Crystal structure of bis­{μ-(*E*)-2-[(2-oxido­phenyl­imino)­meth­yl]quinolin-8-olato-κ^4^ *O*,*N*,*N*′,*O*′}bis­[di­butyl­tin(IV)]

**DOI:** 10.1107/S2056989016018867

**Published:** 2017-01-01

**Authors:** Camacho-Camacho Carlos, Ortiz-Pastrana Naytzé, Garza-Ortiz Ariadna, Rojas-Oviedo Irma

**Affiliations:** a Departamento de Sistemas Biológicos, Universidad Autónoma Metropolitana-Unidad Xochimilco, Calzada de Hueso 1100, Colonia Villa Quietud, 04960, Coyoacán, México, CDMX, Mexico; b Departamento de Química, Cinvestav, Av. Instituto Politécnico Nacional 2508, Col. San Pedro Zacatenco, 07360, Delegación Gustavo A. Madero, México, CDMX, Mexico; c Universidad de la Costa, Carretera al Libramiento Paraje de Las Pulgas S/N, Santiago Pinotepa Nacional, Distrito Jamiltepec, C.P. 71600, Oaxaca, Mexico

**Keywords:** crystal structure, 8-quinolino­lates, sevenfold coordination, di­alkyl­ditin(IV) compound

## Abstract

Condensation of 8-hy­droxy­quinoline-2-carbaldehyde with 2-amino­phenol gave the (*E*)-2-[(2-hy­droxy­phenyl­imino)­meth­yl]quinolin-8-ol derivative that reacted with di-*n*-butyl­tin oxide with release of H_2_O to yield the chelate title complex, [Sn_2_(C_4_H_9_)_4_(C_16_H_10_N_2_O_2_)_2_]. The compound crystallizes in the triclinic space group *P*-1, with two independent centrosymmetric dimers in the unit cell. Each features a typical pincer-type structure where the dianionic ligand is tetra­dentate, coordinating to the central tin atom through both phenolate oxygen atoms, as well as through the quinoline and imine N atoms. Each metal atom adopts a distorted penta­gonal–bipyramidal SnC_2_N_2_O_3_ coordination arising from the *N*,*N*′,*O*,*O*′-tetra­dentate deprotonated Schiff base, one bridging phenolate O atom of the neighbouring ligand and two butyl groups in the axial sites.

## Abstract 2

The title compound crystallizes with two independent centrosymmetric dimers in the unit cell, each featuring a typical pincer-type structure where the dianionic ligand is tetra­dentate, coordinating to the central tin atom through both phenolate oxygen atoms, as well as through the quinoline and imine N atoms. Each metal atom adopts a distorted penta­gonal–bipyramidal SnC_2_N_2_O_3_ coordination arising from the *N*,*N*′,*O*,*O*′-tetra­dentate deprotonated Schiff base and two butyl groups in the axial sites.

## Chemical context   

We are inter­ested in the preparation of organometallic tin compounds derived from biologically active mol­ecules. One of the aims of our research is the structural analysis, particularly their coordination modes which has influence on their biologi­cal effects. The title compound (I) includes a ligand derived from quinoline and 2-amino­phenol. It has been reported that quinoline-bearing structures show broad bio­logical activities such as anti­fungal (Musiol *et al.*, 2006 ▸), anti­malarial (Nasveld & Kitchener, 2005 ▸), and anti­tumor (Rasoul-Amini *et al.*, 2006 ▸). The activity of bis-quinolines as anti­leshmanial agents has also been reported through *in vitro* and *in vivo* studies (Palit *et al.*, 2009 ▸). More recently, it has been shown that quinoline-based thio­semicarbazones present anti­tumor efficacy involving an iron chelation mechanism (Serda *et al.*, 2012 ▸). In addition, Schiff bases derived from 8-hy­droxy­quinoline and its derivatives are well known for their ability towards the complexation of many metals (Charles & Perrotto, 1964 ▸; Corcé *et al.*, 2014 ▸; Albrecht *et al.*, 2005 ▸, 2007 ▸). We report here the crystal structure of a new tin(IV) complex derived from a ligand produced from the 1:1 condensation of 8-hy­droxy­quinoline-2-carboxaldehyde and 2-amino­phenol. The Schiff base H_2_ *L* produced was complexed with di-*n*–butyl­tin oxide to give the title compound (I), [Sn_2_(C_4_H_9_)_4_(C_16_H_10_N_2_O_2_)_2_].
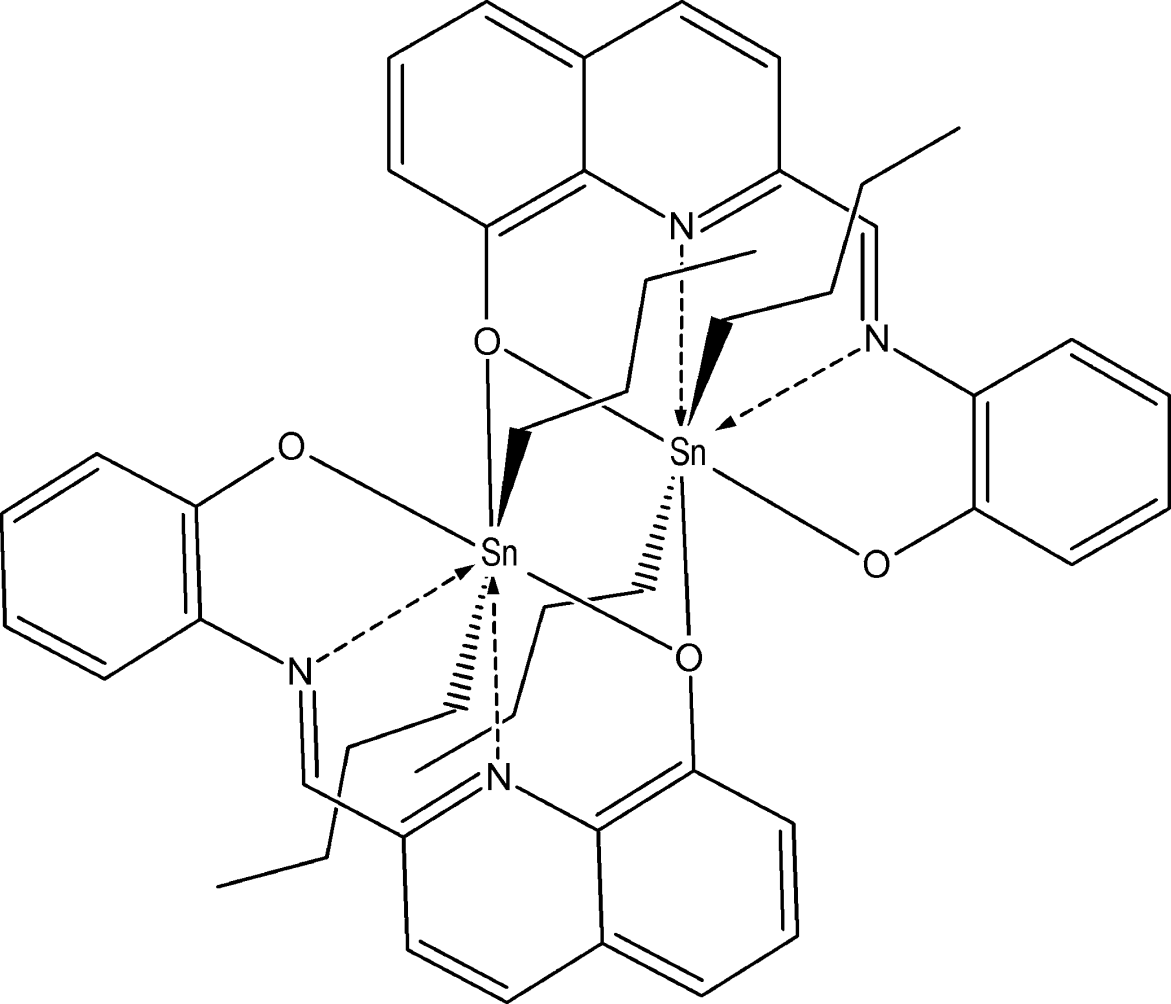


## Structural commentary   

**Figure 1:**
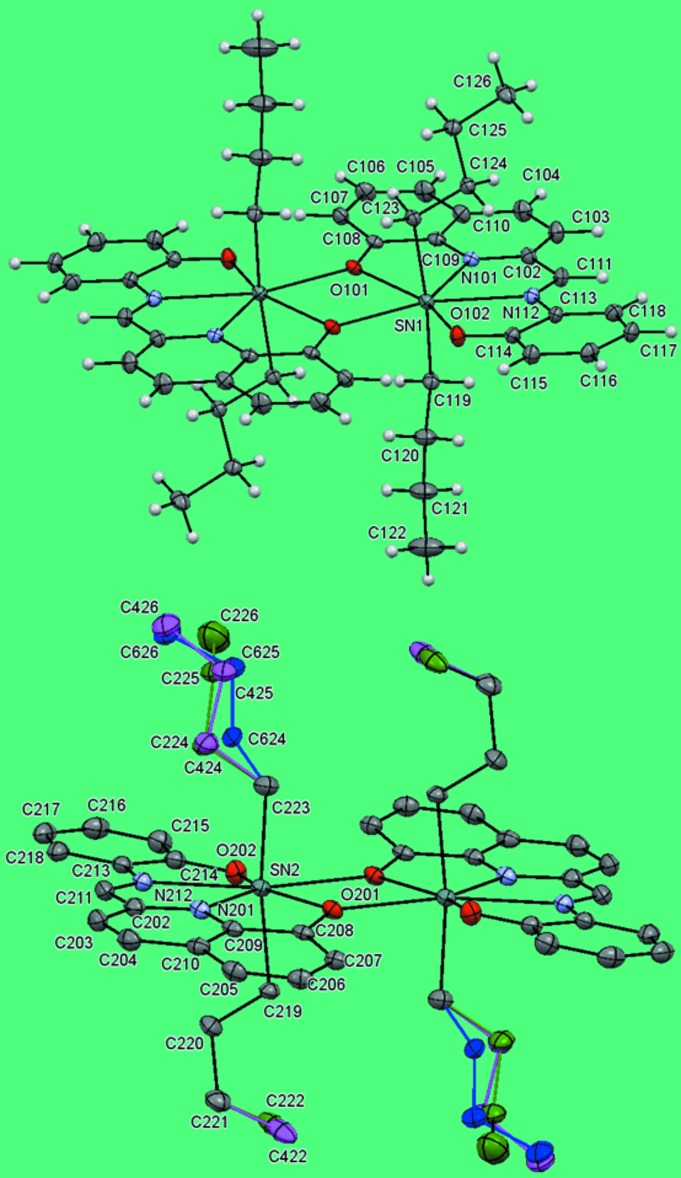
The mol­ecular structures of the two independent molecules in the title compound (I). Displacement ellipsoids are drawn at the 50% probability level. The major part of the disordered *n*-butyl group is shown with green bonds, while the minor components are shown with magenta and blue bonds. H atoms are omitted for clarity.

The mol­ecular structures of the two independent molecules in the compound (I) are shown in Fig. 1 ▸. The structure consists of an isolated homobimetallic dimer located on crystallographic inversion centres so that two independent *n*-Bu_2_Sn*L* units [mol­ecule 1: Sn1 to C126 and mol­ecule 2: Sn2 to C226 (with the *n*-butyl-disordered C119–C222 atoms being slightly disordered at the terminal methyl end and the C223–C226 atoms heavily disordered with a threefold splitting)] comprise the asymmetric unit. The dimerization of these monomeric units occurs through the quinolin-8-olate group oxygen atoms, leading to a central four membered (SnO)_2_ ring with a metal–metal separations Sn1⋯ Sn1(1 − *x*, 1 − *y*, −*z*) 3.9593 (5) Å and Sn2⋯ Sn2(1 − *x*, −*y*, 1 − *z*) = 4.0132 (5) Å.

The (*E*)-2-(oxido­phenyl­imino)­meth­yl)quinolin-8-olate ligands are essentially planar and act as *N*,*N*′,*O*,*O*′-tetra­dentate ligands, forming a lozenge-shaped plane with the two *n*-butyl groups bonded to the same Sn atom on opposite sides. These *n*-butyl groups in mol­ecule 1 display an ordered extended conformation while those in mol­ecule 2 display mainly disordered *gauche* conformations, probably due to packing considerations (Fig. 1 ▸). Each of the two symmetry-independent tin atoms exhibits a slightly distorted penta­gonal–bipyramidal coordination geometry with equatorial (*E*)-2-[(oxido­phenyl­imino)meth­yl]quinolin-8-olate ligands and *n*-butyl groups occupying axial positions [C123—Sn1—C119 = 171.11 (12)° and C223—Sn2—C219 = 171.72 (13)° (Fig. 1 ▸). There are four fused rings (three five-membered and one four-membered) formed between the tin atom and the tetra­dentate bridging ligand.

## Supra­molecular features   

**Figure 2:**
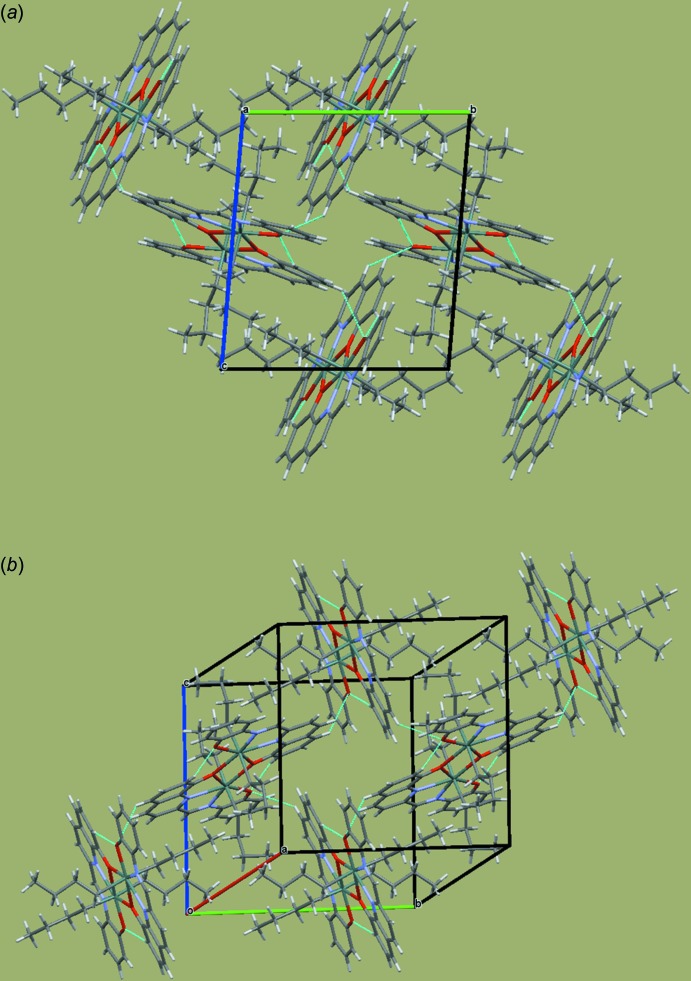
The crystal packing of compound (I): (*a*) viewed down the *a* axis and (*b*) showing the inter­molecular contacts (dashed lines).

The lozenge planes of the two independent mol­ecules are nearly perpendicular [angle between planes = 84.20 (3)°], giving rise to several weak C—H⋯π inter­actions between the *n*-butyl groups attached to the Sn atoms and the aromatic H atoms of the ligand (Table 1 ▸). These inter­actions are complemented by C—H⋯O hydrogen-bonding inter­actions between the adjacent chains (Table 1 ▸, Fig. 2 ▸ *a*). Such inter­actions generate a two-dimensional supra­molecular structure parallel to the *bc* plane (Fig. 2 ▸ *b*).

## Database survey   

There are five examples in the literature of di­alkyl­ditin(IV) compounds with bis­(μ_2_-quinolin-8-olato) ligands (Vafaee *et al.*, 2010 ▸; Basu Baul *et al.*, 2009 ▸). All of these feature an octa­hedral coordination sphere for the tin atoms. The most curious feature of the structure of the title compound is the sevenfold coordination of each Sn atom in the binuclear core, although this coordination number is not unprecedented in the structural chemistry of tin (de Sousa *et al.*, 2009 ▸). Only the diorgano­tin(IV) complexes of pyruvic acid picolino­acyl­hydrazone (Cui *et al.*, 2010 ▸) share the characteristic of being formed by four rings (three five-membered rings and one four-membered ring) and both are centrosymmetric. In pyruvic acid picolino­acyl­hydrazone, the four-membered Sn_2_O_2_ ring shares two edges with two other five-membered rings. Meanwhile, in the title compound each ring is only fused to one another, giving rise to a more extended structure. Previously, representative elements (Sun *et al.*, 2011 ▸), transition (Anitha *et al.*, 2015 ▸; García-Santos *et al.*, 2009 ▸; Yan *et al.*, 2014 ▸) and lanthanide (Zhang *et al.*, 2012 ▸, 2015 ▸) metal complexes of [(imino)­meth­yl]quinolin-8-olato derivatives have been reported, and only in the case of the lanthanide complexes is the nitro­gen atom of the imine group involved in the ligand coordination. So, to the best of our knowledge, the title compound is the first example of a [(imino)­meth­yl]quinolin-8-olato derivative with the ligand using the full possible denticity.

## Synthesis and crystallization   

3-Hy­droxy­quinoline-2-carboxaldehyde, 2-amino­phenol, di-*n*-butyl­tin(IV) oxide and solvents were purchased from Aldrich and used without further purification. Elemental analysis were performed using an Eager 300 analyzer. The infrared spectra were recorded on Perkin Elmer 1600 FT spectrometer in the 4000–400 cm^−1^ range. Melting points were measured on a Fisher–Johns melting-point apparatus and are uncorrected. ^119^Sn spectra were recorded with a Bruker AVANCE-II, 300 MHz NMR spectrometer operating at 111.81 MHz and using a 4mm CP-MAS probe. NMR ^119^Sn chemical shift referencing is toward tetra­methyl­tin.

**Figure 3:**
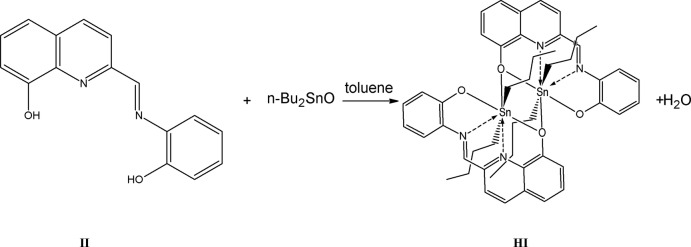
Synthesis pathway.

Compound (I). (Synthesis pathway is shown in Fig. 3 ▸.) Equimolar qu­anti­ties of 2-[*N*-(2-hy­droxy­phen­yl)carb­oxim­ido­yl]quinolin-8-ol (II) (0.378 mmol) and di-*n*-butyl­tin oxide (0.378 mmol) were dissolved in toluene in a 100 ml flask equipped with a Dean–Stark funnel. This mixture was refluxed for 1.5 h. After refluxing, the solvent was distilled. The red crystalline product was recrystallized from a mixture of 2,3-di­chloro­butane/hexane 3/1. M.p. 468–471 K. Yield 69.4% The dark-red crystalline product was characterized by elemental analysis, calculated for C_24_H_28_N_2_O_2_Sn 0.3H_2_O, C 57.55, H 5.61, N 5.59. Found C 57.30, H 5.32, N 5.51. RMN ^119^Sn solid state: −462.57 ppm. IR (KBr; *s* = strong, *m* = medium, *w* = weak) 3074*w*, 2954*s*, 2922*s*, 2867m, 2854*m*, 1591*m*, 1581*m*, 1523*m*, 1504*m*, 1470*s*, 1446*m*, 1429*m*, 1377*w*, 1342*m*, 1329*s*, 1308*m*, 1286*m*, 1188*w*, 1142*m*, 1135*m*, 1094*w*, 907*w*, 881w, 839*w*, 752*m*, 742*m*, 549*w*, 468*w*.

## Refinement   

Crystal data, data collection and structure refinement details are summarized in Table 2 ▸. H atoms were placed in calculated positions (C—H = 0.95–0.99 Å) and treated in the riding approximation with isotropic displacement parameters set at 1.2–1.5 times the *U*_eq_ value of the parent atom. The *n*-butyl groups in molecule 2 display some degree of orientational disorder, which was modeled into two orientations using geometrical (SADI, SIMU) and ADP (SIMU, DELU) restraints.

## Supplementary Material

Crystal structure: contains datablock(s) I, Compound_I. DOI: 10.1107/S2056989016018867/bg2598sup1.cif

Structure factors: contains datablock(s) I. DOI: 10.1107/S2056989016018867/bg2598Isup2.hkl

Supporting information file. DOI: 10.1107/S2056989016018867/bg2598Isup3.cdx

Supporting information file. DOI: 10.1107/S2056989016018867/bg2598Isup4.cdx

CCDC reference: 1519148

Additional supporting information: crystallographic information; 3D view; checkCIF report

## Figures and Tables

**Table 1:** Hydrogen-bond geometry (Å, °) *Cg*2, *Cg*3, *Cg*4 and *Cg*5 are the centroids of the C205–C210, C213–C218, N101/C104/C109C110 and C105–C110 rings, respectively.

*D*—H⋯*A*	*D*—H	H⋯*A*	*D*⋯*A*	*D*—H⋯*A*
C107—H107⋯O102^i^	0.95	2.09	2.908 (4)	144
C207—H207⋯O202^ii^	0.95	2.18	2.972 (4)	140
C105—H105⋯*Cg*2	0.95	2.71	3.609 (3)	159
C116—H116⋯*Cg*3^iii^	0.95	2.94	3.704 (3)	139
C125—H132⋯*Cg*2^iv^	0.99	2.77	3.602 (3)	142
C204—H204⋯*Cg*5^v^	0.95	2.77	3.700 (4)	165
C222—H226⋯*Cg*4^ii^	0.98	2.69	3.613 (14)	157
C422—H426⋯*Cg*5^ii^	0.98	2.94	3.77 (3)	142

Symmetry codes: (i) 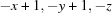; (ii) 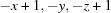; (iii) 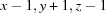; (iv) 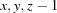; (v) 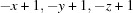.

**Table 2:** Experimental details

Crystal data	
Chemical formula	[Sn_2_(C_4_H_9_)_4_(C_16_H_10_N_2_O_2_)_2_]
*M*_r_	990.34
Crystal system, space group	Triclinic, *P* 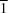
Temperature (K)	123
*a*, *b*, *c* (Å)	13.4874 (7), 13.7517 (8), 13.8397 (8)
α, β, γ (°)	89.480 (1), 80.345 (1), 60.858 (1)
*V* (Å^3^)	2202.4 (2)
*Z*	2
Radiation type	Mo *K*α
μ (mm^−1^)	1.18
Crystal size (mm)	0.33 × 0.23 × 0.22

Data collection	
Diffractometer	Bruker SMART APEX CCD
Absorption correction	Multi-scan (*SADABS*; Bruker 2012 ▸)
*T*_min_, *T*_max_	0.648, 0.819
No. of measured, independent and observed [*I* > 2σ(*I*)] reflections	16941, 8017, 6885
*R*_int_	0.028
(sin θ/λ)_max_ (Å^−1^)	0.604

Refinement	
*R*[*F* ^2^ > 2σ(*F* ^2^)], *wR*(*F* ^2^), *S*	0.032, 0.077, 1.04
No. of reflections	8017
No. of parameters	591
No. of restraints	254
H-atom treatment	H-atom parameters constrained
Δρ_max_, Δρ_min_ (e Å^−3^)	0.77, −0.70

Computer programs: *APEX2* and *SAINT* (Bruker, 2012 ▸), *SHELXS2012* (Sheldrick, 2008 ▸), *SHELXL2014* (Sheldrick, 2015 ▸), *Mercury* (Macrae *et al.*, 2008 ▸) and *publCIF* (Westrip, 2010 ▸).

## supplementary crystallographic information

### Crystal data

**Table:**

[Sn_2_(C_4_H_9_)_4_(C_16_H_10_N_2_O_2_)_2_]	*F*(000) = 1008
*M**_r_* = 990.34	*D*_x_ = 1.493 Mg m^−^^3^
Triclinic, *P*1	Melting point = 198–195 K
*a* = 13.4874 (7) Å	Mo *K*α radiation, λ = 0.71073 Å
*b* = 13.7517 (8) Å	Cell parameters from 9912 reflections
*c* = 13.8397 (8) Å	θ = 2.4–25.4°
α = 89.480 (1)°	µ = 1.18 mm^−^^1^
β = 80.345 (1)°	*T* = 123 K
γ = 60.858 (1)°	Block, dark-red
*V* = 2202.4 (2) Å^3^	0.33 × 0.23 × 0.22 mm
*Z* = 2	

### Data collection

**Table:**

Bruker SMART APEX CCD diffractometer	8017 independent reflections
Radiation source: fine-focus sealed tube	6885 reflections with *I* > 2σ(*I*)
Graphite monochromator	*R*_int_ = 0.028
Detector resolution: 8.333 pixels mm^-1^	θ_max_ = 25.4°, θ_min_ = 1.7°
ω–scans	*h* = −16→16
Absorption correction: multi-scan (SADABS; Bruker, 2012)	*k* = −16→16
*T*_min_ = 0.648, *T*_max_ = 0.819	*l* = −16→16
16941 measured reflections	

### Refinement

**Table:**

Refinement on *F*^2^	Primary atom site location: structure-invariant direct methods
Least-squares matrix: full	Secondary atom site location: difference Fourier map
*R*[*F*^2^ > 2σ(*F*^2^)] = 0.032	Hydrogen site location: inferred from neighbouring sites
*wR*(*F*^2^) = 0.077	H-atom parameters constrained
*S* = 1.04	*w* = 1/[σ^2^(*F*_o_^2^) + (0.0356*P*)^2^ + 1.1148*P*] where *P* = (*F*_o_^2^ + 2*F*_c_^2^)/3
8017 reflections	(Δ/σ)_max_ = 0.001
591 parameters	Δρ_max_ = 0.77 e Å^−^^3^
254 restraints	Δρ_min_ = −0.70 e Å^−^^3^

### Special details

**Table:**

Geometry. All esds (except the esd in the dihedral angle between two l.s. planes) are estimated using the full covariance matrix. The cell esds are taken into account individually in the estimation of esds in distances, angles and torsion angles; correlations between esds in cell parameters are only used when they are defined by crystal symmetry. An approximate (isotropic) treatment of cell esds is used for estimating esds involving l.s. planes.
Refinement. *n*-butyl groups in molecule 2 display some degree of orientational disorder which was modeled into two orientations using geometrical (SADI, SIMU) and ADP (SIMU, DELU) restrains.

### Fractional atomic coordinates and isotropic or equivalent isotropic displacement parameters (Å^2^)

**Table:**

	*x*	*y*	*z*	*U*_iso_*/*U*_eq_	Occ. (<1)
Sn1	0.33665 (2)	0.54296 (2)	0.00166 (2)	0.02140 (7)	
O101	0.50270 (18)	0.44999 (16)	0.08935 (14)	0.0222 (5)	
O102	0.24816 (18)	0.61885 (19)	−0.11825 (15)	0.0293 (5)	
N101	0.2869 (2)	0.4844 (2)	0.15872 (18)	0.0221 (5)	
C102	0.1801 (3)	0.5028 (3)	0.1907 (2)	0.0258 (7)	
C103	0.1468 (3)	0.4711 (3)	0.2820 (2)	0.0326 (8)	
H103	0.0695	0.4864	0.3035	0.039*	
C104	0.2283 (3)	0.4177 (3)	0.3390 (2)	0.0332 (8)	
H104	0.2080	0.3938	0.4001	0.040*	
C105	0.4302 (3)	0.3403 (3)	0.3623 (2)	0.0355 (8)	
H105	0.4138	0.3181	0.4255	0.043*	
C106	0.5404 (3)	0.3170 (3)	0.3225 (2)	0.0336 (8)	
H106	0.6003	0.2771	0.3587	0.040*	
C107	0.5681 (3)	0.3501 (3)	0.2302 (2)	0.0284 (7)	
H107	0.6461	0.3299	0.2047	0.034*	
C108	0.4837 (3)	0.4117 (2)	0.1752 (2)	0.0215 (6)	
C109	0.3688 (3)	0.4330 (2)	0.2150 (2)	0.0229 (7)	
C110	0.3424 (3)	0.3974 (3)	0.3083 (2)	0.0279 (7)	
C111	0.1004 (3)	0.5552 (3)	0.1229 (2)	0.0277 (7)	
H111	0.0219	0.5728	0.1402	0.033*	
N112	0.1394 (2)	0.5769 (2)	0.03934 (18)	0.0244 (6)	
C113	0.0774 (3)	0.6210 (2)	−0.0369 (2)	0.0228 (7)	
C114	0.1428 (3)	0.6403 (3)	−0.1182 (2)	0.0249 (7)	
C115	0.0896 (3)	0.6832 (3)	−0.1998 (2)	0.0304 (7)	
H115	0.1305	0.6978	−0.2555	0.036*	
C116	−0.0213 (3)	0.7040 (3)	−0.1995 (2)	0.0322 (8)	
H116	−0.0557	0.7324	−0.2554	0.039*	
C117	−0.0840 (3)	0.6841 (3)	−0.1185 (3)	0.0329 (8)	
H117	−0.1608	0.6998	−0.1188	0.039*	
C118	−0.0336 (3)	0.6416 (3)	−0.0381 (2)	0.0308 (7)	
H118	−0.0751	0.6262	0.0167	0.037*	
C119	0.2880 (3)	0.6950 (3)	0.0836 (2)	0.0264 (7)	
H119	0.2183	0.7134	0.1337	0.032*	
H120	0.3507	0.6816	0.1193	0.032*	
C120	0.2628 (3)	0.7965 (3)	0.0263 (3)	0.0373 (8)	
H121	0.1946	0.8162	−0.0040	0.045*	
H122	0.3294	0.7777	−0.0274	0.045*	
C121	0.2398 (4)	0.8973 (3)	0.0910 (3)	0.0505 (11)	
H123	0.1814	0.9086	0.1500	0.061*	
H124	0.3120	0.8809	0.1134	0.061*	
C122	0.1983 (5)	1.0028 (4)	0.0407 (4)	0.0771 (16)	
H125	0.2590	0.9952	−0.0138	0.116*	
H126	0.1794	1.0649	0.0877	0.116*	
H127	0.1291	1.0177	0.0151	0.116*	
C123	0.4060 (3)	0.3797 (2)	−0.0672 (2)	0.0249 (7)	
H128	0.4497	0.3757	−0.1335	0.030*	
H129	0.4617	0.3258	−0.0289	0.030*	
C124	0.3188 (3)	0.3423 (2)	−0.0782 (2)	0.0256 (7)	
H130	0.2770	0.3422	−0.0122	0.031*	
H131	0.2615	0.3962	−0.1154	0.031*	
C125	0.3767 (3)	0.2261 (2)	−0.1314 (2)	0.0284 (7)	
H132	0.4183	0.2267	−0.1973	0.034*	
H133	0.4346	0.1727	−0.0944	0.034*	
C126	0.2930 (3)	0.1856 (3)	−0.1432 (3)	0.0377 (9)	
H134	0.2544	0.1808	−0.0782	0.056*	
H135	0.3352	0.1116	−0.1795	0.056*	
H136	0.2351	0.2381	−0.1796	0.056*	
Sn2	0.65168 (2)	−0.03232 (2)	0.53350 (2)	0.02447 (7)	
O201	0.44927 (19)	0.10973 (17)	0.53347 (15)	0.0264 (5)	
O202	0.80224 (19)	−0.20279 (18)	0.52234 (17)	0.0343 (5)	
N201	0.6075 (2)	0.1500 (2)	0.59076 (19)	0.0276 (6)	
C202	0.6895 (3)	0.1671 (3)	0.6160 (2)	0.0314 (8)	
C203	0.6655 (3)	0.2733 (3)	0.6513 (2)	0.0368 (8)	
H203	0.7259	0.2847	0.6654	0.044*	
C204	0.5549 (3)	0.3597 (3)	0.6651 (3)	0.0374 (9)	
H204	0.5381	0.4318	0.6889	0.045*	
C205	0.3474 (3)	0.4253 (3)	0.6647 (2)	0.0351 (8)	
H205	0.3241	0.4986	0.6904	0.042*	
C206	0.2679 (3)	0.3980 (3)	0.6467 (2)	0.0348 (8)	
H206	0.1884	0.4527	0.6635	0.042*	
C207	0.2971 (3)	0.2926 (3)	0.6043 (2)	0.0298 (7)	
H207	0.2378	0.2782	0.5927	0.036*	
C208	0.4127 (3)	0.2090 (3)	0.5790 (2)	0.0251 (7)	
C209	0.4969 (3)	0.2348 (3)	0.6039 (2)	0.0263 (7)	
C210	0.4652 (3)	0.3430 (3)	0.6444 (2)	0.0308 (8)	
C211	0.8030 (3)	0.0691 (3)	0.6054 (2)	0.0329 (8)	
H211	0.8669	0.0743	0.6196	0.039*	
N212	0.8148 (2)	−0.0250 (2)	0.57622 (19)	0.0292 (6)	
C213	0.9182 (3)	−0.1272 (3)	0.5607 (2)	0.0302 (7)	
C214	0.9039 (3)	−0.2176 (3)	0.5307 (2)	0.0318 (8)	
C215	1.0036 (3)	−0.3238 (3)	0.5114 (3)	0.0393 (9)	
H215	0.9974	−0.3865	0.4924	0.047*	
C216	1.1101 (3)	−0.3377 (3)	0.5200 (3)	0.0427 (9)	
H216	1.1765	−0.4097	0.5055	0.051*	
C217	1.1216 (3)	−0.2478 (3)	0.5495 (3)	0.0427 (9)	
H217	1.1953	−0.2589	0.5559	0.051*	
C218	1.0259 (3)	−0.1428 (3)	0.5695 (2)	0.0376 (9)	
H218	1.0336	−0.0812	0.5893	0.045*	
C219	0.5850 (3)	−0.0562 (3)	0.6769 (2)	0.0279 (7)	
H219	0.5123	0.0135	0.7027	0.033*	
H220	0.5646	−0.1157	0.6704	0.033*	
C220	0.6614 (3)	−0.0867 (3)	0.7527 (2)	0.0401 (9)	
H221	0.6906	−0.0332	0.7538	0.048*	
H222	0.7290	−0.1620	0.7327	0.048*	
C221	0.6011 (3)	−0.0867 (3)	0.8566 (3)	0.0440 (9)	
H223	0.6550	−0.1014	0.9024	0.053*	0.32 (3)
H224	0.5336	−0.0114	0.8766	0.053*	0.32 (3)
H423	0.6623	−0.1311	0.8947	0.053*	0.68 (3)
H424	0.5602	−0.0088	0.8866	0.053*	0.68 (3)
C222	0.5613 (15)	−0.1701 (12)	0.8667 (8)	0.050 (3)	0.68 (3)
H225	0.5289	−0.1689	0.9358	0.075*	0.68 (3)
H226	0.6269	−0.2447	0.8442	0.075*	0.68 (3)
H227	0.5020	−0.1519	0.8267	0.075*	0.68 (3)
C422	0.5172 (19)	−0.128 (2)	0.8727 (19)	0.050 (3)	0.32 (3)
H425	0.4818	−0.1156	0.9426	0.075*	0.32 (3)
H426	0.5574	−0.2086	0.8525	0.075*	0.32 (3)
H427	0.4570	−0.0885	0.8337	0.075*	0.32 (3)
C223	0.6954 (3)	−0.0042 (3)	0.3850 (2)	0.0392 (9)	
H228	0.7017	−0.0653	0.3425	0.047*	0.432 (3)
H229	0.6296	0.0660	0.3707	0.047*	0.432 (3)
H428	0.7090	−0.0697	0.3439	0.047*	0.400 (3)
H429	0.6254	0.0607	0.3692	0.047*	0.400 (3)
H628	0.6448	−0.0150	0.3472	0.047*	0.169 (3)
H629	0.6749	0.0753	0.3837	0.047*	0.169 (3)
C224	0.8025 (13)	0.0037 (19)	0.3537 (8)	0.041 (3)	0.432 (3)
H230	0.8660	−0.0584	0.3798	0.050*	0.432 (3)
H231	0.7899	0.0743	0.3849	0.050*	0.432 (3)
C225	0.8419 (9)	0.0004 (9)	0.2443 (9)	0.047 (3)	0.432 (3)
H232	0.8704	0.0544	0.2328	0.057*	0.432 (3)
H233	0.7741	0.0257	0.2120	0.057*	0.432 (3)
C226	0.9355 (10)	−0.1126 (9)	0.1954 (8)	0.079 (3)	0.432 (3)
H234	1.0060	−0.1354	0.2219	0.118*	0.432 (3)
H235	0.9515	−0.1078	0.1243	0.118*	0.432 (3)
H236	0.9098	−0.1677	0.2083	0.118*	0.432 (3)
C424	0.7940 (15)	0.016 (2)	0.3493 (9)	0.041 (3)	0.400 (3)
H430	0.8657	−0.0467	0.3650	0.049*	0.400 (3)
H431	0.7799	0.0852	0.3846	0.049*	0.400 (3)
C425	0.8115 (10)	0.0274 (12)	0.2391 (10)	0.043 (2)	0.400 (3)
H432	0.8148	−0.0371	0.2045	0.051*	0.400 (3)
H433	0.7437	0.0959	0.2248	0.051*	0.400 (3)
C426	0.9192 (10)	0.0332 (12)	0.1987 (9)	0.057 (3)	0.400 (3)
H434	0.9198	0.0930	0.2361	0.086*	0.400 (3)
H435	0.9205	0.0486	0.1294	0.086*	0.400 (3)
H436	0.9874	−0.0383	0.2044	0.086*	0.400 (3)
C624	0.8183 (9)	−0.0732 (14)	0.3299 (12)	0.036 (3)	0.169 (3)
H630	0.8715	−0.0713	0.3702	0.043*	0.169 (3)
H631	0.8365	−0.1518	0.3202	0.043*	0.169 (3)
C625	0.839 (2)	−0.032 (3)	0.2310 (15)	0.044 (3)	0.169 (3)
H632	0.8488	−0.0871	0.1793	0.052*	0.169 (3)
H633	0.7686	0.0390	0.2255	0.052*	0.169 (3)
C626	0.941 (2)	−0.013 (3)	0.210 (2)	0.052 (5)	0.169 (3)
H634	0.9177	0.0624	0.2358	0.077*	0.169 (3)
H635	0.9690	−0.0223	0.1391	0.077*	0.169 (3)
H636	1.0025	−0.0678	0.2423	0.077*	0.169 (3)

### Atomic displacement parameters (Å^2^)

**Table:**

	*U*^11^	*U*^22^	*U*^33^	*U*^12^	*U*^13^	*U*^23^
Sn1	0.02267 (13)	0.02310 (12)	0.02163 (12)	−0.01351 (10)	−0.00488 (9)	0.00249 (8)
O101	0.0277 (12)	0.0252 (11)	0.0189 (11)	−0.0168 (10)	−0.0054 (9)	0.0056 (9)
O102	0.0249 (12)	0.0406 (14)	0.0267 (12)	−0.0183 (11)	−0.0086 (10)	0.0103 (10)
N101	0.0240 (14)	0.0216 (13)	0.0217 (13)	−0.0121 (12)	−0.0044 (11)	0.0026 (10)
C102	0.0258 (17)	0.0292 (17)	0.0244 (17)	−0.0156 (15)	−0.0027 (13)	0.0018 (13)
C103	0.0300 (19)	0.044 (2)	0.0237 (17)	−0.0197 (17)	0.0002 (14)	0.0028 (15)
C104	0.038 (2)	0.043 (2)	0.0184 (17)	−0.0220 (18)	0.0005 (14)	0.0063 (14)
C105	0.041 (2)	0.045 (2)	0.0236 (18)	−0.0236 (18)	−0.0075 (15)	0.0132 (15)
C106	0.040 (2)	0.040 (2)	0.0294 (18)	−0.0231 (18)	−0.0168 (16)	0.0164 (15)
C107	0.0300 (18)	0.0306 (18)	0.0302 (18)	−0.0187 (16)	−0.0077 (14)	0.0077 (14)
C108	0.0282 (17)	0.0203 (15)	0.0212 (16)	−0.0155 (14)	−0.0058 (13)	0.0011 (12)
C109	0.0294 (18)	0.0216 (16)	0.0200 (16)	−0.0140 (14)	−0.0059 (13)	0.0016 (12)
C110	0.0332 (19)	0.0328 (18)	0.0209 (16)	−0.0190 (16)	−0.0039 (14)	0.0055 (13)
C111	0.0214 (17)	0.0325 (18)	0.0275 (18)	−0.0130 (15)	−0.0014 (13)	0.0017 (14)
N112	0.0236 (14)	0.0250 (14)	0.0254 (14)	−0.0121 (12)	−0.0058 (11)	0.0029 (11)
C113	0.0192 (16)	0.0227 (16)	0.0254 (17)	−0.0092 (13)	−0.0047 (13)	0.0022 (13)
C114	0.0233 (17)	0.0233 (16)	0.0283 (17)	−0.0111 (14)	−0.0065 (13)	0.0027 (13)
C115	0.0274 (18)	0.0354 (19)	0.0275 (18)	−0.0148 (16)	−0.0057 (14)	0.0079 (14)
C116	0.0298 (19)	0.0340 (19)	0.0328 (19)	−0.0130 (16)	−0.0147 (15)	0.0068 (15)
C117	0.0209 (18)	0.0337 (19)	0.041 (2)	−0.0103 (15)	−0.0085 (15)	0.0034 (15)
C118	0.0276 (18)	0.0291 (18)	0.0342 (19)	−0.0127 (15)	−0.0063 (15)	0.0057 (14)
C119	0.0256 (17)	0.0290 (17)	0.0250 (17)	−0.0138 (15)	−0.0045 (13)	−0.0021 (13)
C120	0.047 (2)	0.0270 (18)	0.040 (2)	−0.0185 (17)	−0.0106 (17)	0.0020 (15)
C121	0.070 (3)	0.032 (2)	0.053 (3)	−0.027 (2)	−0.016 (2)	0.0006 (18)
C122	0.117 (5)	0.038 (3)	0.077 (4)	−0.037 (3)	−0.024 (3)	0.007 (2)
C123	0.0244 (17)	0.0274 (17)	0.0246 (17)	−0.0146 (14)	−0.0027 (13)	0.0016 (13)
C124	0.0251 (17)	0.0282 (17)	0.0267 (17)	−0.0156 (15)	−0.0043 (13)	0.0016 (13)
C125	0.0296 (18)	0.0276 (17)	0.0284 (18)	−0.0144 (15)	−0.0051 (14)	−0.0005 (14)
C126	0.042 (2)	0.040 (2)	0.036 (2)	−0.0268 (18)	0.0000 (16)	−0.0081 (16)
Sn2	0.02645 (13)	0.02462 (13)	0.02094 (12)	−0.01130 (10)	−0.00517 (9)	0.00264 (9)
O201	0.0325 (13)	0.0209 (11)	0.0238 (11)	−0.0111 (10)	−0.0065 (9)	−0.0001 (9)
O202	0.0291 (13)	0.0284 (12)	0.0404 (14)	−0.0093 (11)	−0.0099 (11)	0.0004 (10)
N201	0.0349 (16)	0.0263 (14)	0.0223 (14)	−0.0151 (13)	−0.0067 (12)	0.0030 (11)
C202	0.039 (2)	0.0346 (19)	0.0247 (17)	−0.0209 (17)	−0.0051 (15)	0.0042 (14)
C203	0.046 (2)	0.043 (2)	0.0313 (19)	−0.030 (2)	−0.0065 (16)	−0.0013 (16)
C204	0.052 (2)	0.034 (2)	0.0317 (19)	−0.0258 (19)	−0.0042 (17)	−0.0025 (15)
C205	0.045 (2)	0.0242 (17)	0.0299 (19)	−0.0133 (17)	−0.0050 (16)	−0.0040 (14)
C206	0.036 (2)	0.0262 (18)	0.0311 (19)	−0.0089 (16)	−0.0004 (15)	−0.0012 (14)
C207	0.035 (2)	0.0280 (18)	0.0249 (17)	−0.0151 (16)	−0.0040 (14)	0.0018 (14)
C208	0.0341 (19)	0.0236 (16)	0.0150 (15)	−0.0124 (15)	−0.0042 (13)	0.0031 (12)
C209	0.038 (2)	0.0248 (17)	0.0175 (16)	−0.0172 (16)	−0.0044 (14)	0.0030 (12)
C210	0.044 (2)	0.0256 (17)	0.0222 (17)	−0.0185 (16)	−0.0016 (15)	0.0004 (13)
C211	0.038 (2)	0.044 (2)	0.0264 (18)	−0.0260 (18)	−0.0099 (15)	0.0043 (15)
N212	0.0282 (15)	0.0348 (16)	0.0237 (14)	−0.0148 (13)	−0.0052 (12)	0.0034 (12)
C213	0.0311 (19)	0.0365 (19)	0.0200 (17)	−0.0146 (16)	−0.0041 (14)	0.0027 (14)
C214	0.0285 (19)	0.0368 (19)	0.0238 (17)	−0.0116 (16)	−0.0038 (14)	0.0029 (14)
C215	0.034 (2)	0.038 (2)	0.036 (2)	−0.0101 (17)	−0.0071 (16)	0.0017 (16)
C216	0.028 (2)	0.044 (2)	0.035 (2)	−0.0026 (17)	−0.0034 (16)	0.0061 (17)
C217	0.026 (2)	0.062 (3)	0.034 (2)	−0.0158 (19)	−0.0083 (16)	0.0142 (18)
C218	0.034 (2)	0.052 (2)	0.0308 (19)	−0.0231 (19)	−0.0078 (16)	0.0122 (17)
C219	0.0267 (18)	0.0338 (18)	0.0205 (16)	−0.0126 (15)	−0.0055 (13)	0.0070 (13)
C220	0.044 (2)	0.055 (2)	0.0287 (19)	−0.027 (2)	−0.0157 (16)	0.0115 (17)
C221	0.049 (2)	0.050 (2)	0.032 (2)	−0.021 (2)	−0.0150 (17)	0.0123 (17)
C222	0.087 (7)	0.041 (6)	0.034 (3)	−0.038 (6)	−0.022 (5)	0.013 (5)
C422	0.087 (7)	0.041 (6)	0.034 (3)	−0.038 (6)	−0.022 (5)	0.013 (5)
C223	0.043 (2)	0.044 (2)	0.0276 (19)	−0.0207 (19)	−0.0035 (16)	0.0039 (16)
C224	0.042 (4)	0.052 (5)	0.033 (3)	−0.025 (4)	−0.011 (3)	0.021 (3)
C225	0.050 (4)	0.062 (5)	0.035 (3)	−0.033 (4)	−0.005 (4)	0.015 (4)
C226	0.074 (6)	0.085 (6)	0.068 (5)	−0.033 (5)	−0.011 (5)	−0.007 (5)
C424	0.040 (4)	0.051 (5)	0.032 (3)	−0.022 (4)	−0.011 (3)	0.013 (4)
C425	0.048 (4)	0.054 (5)	0.033 (3)	−0.030 (4)	−0.010 (4)	0.014 (4)
C426	0.065 (6)	0.079 (7)	0.047 (5)	−0.050 (5)	−0.011 (4)	0.026 (5)
C624	0.043 (5)	0.049 (5)	0.032 (4)	−0.035 (5)	−0.010 (4)	0.012 (4)
C625	0.049 (5)	0.056 (6)	0.035 (4)	−0.034 (5)	−0.008 (4)	0.014 (5)
C626	0.060 (8)	0.067 (9)	0.044 (8)	−0.044 (7)	−0.011 (7)	0.028 (8)

### Geometric parameters (Å, º)

**Table:**

Sn1—C123	2.126 (3)	C203—H203	0.9500
Sn1—C119	2.133 (3)	C204—C210	1.410 (5)
Sn1—O102	2.165 (2)	C204—H204	0.9500
Sn1—O101^i^	2.362 (2)	C205—C206	1.355 (5)
Sn1—N101	2.414 (2)	C205—C210	1.411 (5)
Sn1—N112	2.429 (3)	C205—H205	0.9500
Sn1—O101	2.493 (2)	C206—C207	1.407 (4)
O101—C108	1.331 (3)	C206—H206	0.9500
O101—Sn1^i^	2.3617 (19)	C207—C208	1.398 (5)
O102—C114	1.302 (4)	C207—H207	0.9500
N101—C102	1.329 (4)	C208—C209	1.435 (4)
N101—C109	1.358 (4)	C209—C210	1.424 (4)
C102—C103	1.407 (4)	C211—N212	1.284 (4)
C102—C111	1.458 (4)	C211—H211	0.9500
C103—C104	1.365 (5)	N212—C213	1.401 (4)
C103—H103	0.9500	C213—C218	1.388 (5)
C104—C110	1.412 (5)	C213—C214	1.422 (5)
C104—H104	0.9500	C214—C215	1.410 (5)
C105—C106	1.371 (5)	C215—C216	1.379 (5)
C105—C110	1.400 (5)	C215—H215	0.9500
C105—H105	0.9500	C216—C217	1.391 (5)
C106—C107	1.405 (4)	C216—H216	0.9500
C106—H106	0.9500	C217—C218	1.379 (5)
C107—C108	1.389 (4)	C217—H217	0.9500
C107—H107	0.9500	C218—H218	0.9500
C108—C109	1.437 (4)	C219—C220	1.508 (4)
C109—C110	1.427 (4)	C219—H219	0.9900
C111—N112	1.283 (4)	C219—H220	0.9900
C111—H111	0.9500	C220—C221	1.528 (5)
N112—C113	1.403 (4)	C220—H221	0.9900
C113—C118	1.385 (4)	C220—H222	0.9900
C113—C114	1.416 (4)	C221—C222	1.481 (6)
C114—C115	1.410 (4)	C221—C422	1.485 (9)
C115—C116	1.378 (5)	C221—H223	0.9900
C115—H115	0.9500	C221—H224	0.9900
C116—C117	1.396 (5)	C221—H423	0.9900
C116—H116	0.9500	C221—H424	0.9900
C117—C118	1.377 (5)	C222—H225	0.9800
C117—H117	0.9500	C222—H226	0.9800
C118—H118	0.9500	C222—H227	0.9800
C119—C120	1.517 (4)	C422—H425	0.9800
C119—H119	0.9900	C422—H426	0.9800
C119—H120	0.9900	C422—H427	0.9800
C120—C121	1.527 (5)	C223—C424	1.493 (7)
C120—H121	0.9900	C223—C224	1.494 (7)
C120—H122	0.9900	C223—C624	1.512 (7)
C121—C122	1.492 (6)	C223—H228	0.9900
C121—H123	0.9900	C223—H229	0.9900
C121—H124	0.9900	C223—H428	0.9900
C122—H125	0.9800	C223—H429	0.9900
C122—H126	0.9800	C223—H628	0.9900
C122—H127	0.9800	C223—H629	0.9900
C123—C124	1.523 (3)	C224—C225	1.511 (6)
C123—H128	0.9900	C224—H230	0.9900
C123—H129	0.9900	C224—H231	0.9900
C124—C125	1.522 (4)	C225—C226	1.513 (7)
C124—H130	0.9900	C225—H232	0.9900
C124—H131	0.9900	C225—H233	0.9900
C125—C126	1.513 (4)	C226—H234	0.9800
C125—H132	0.9900	C226—H235	0.9800
C125—H133	0.9900	C226—H236	0.9800
C126—H134	0.9800	C424—C425	1.521 (7)
C126—H135	0.9800	C424—H430	0.9900
C126—H136	0.9800	C424—H431	0.9900
Sn2—C223	2.127 (3)	C425—C426	1.507 (7)
Sn2—C219	2.129 (3)	C425—H432	0.9900
Sn2—O202	2.215 (2)	C425—H433	0.9900
Sn2—N201	2.386 (3)	C426—H434	0.9800
Sn2—O201^ii^	2.387 (2)	C426—H435	0.9800
Sn2—N212	2.419 (3)	C426—H436	0.9800
Sn2—O201	2.465 (2)	C624—C625	1.509 (8)
O201—C208	1.327 (3)	C624—H630	0.9900
O201—Sn2^ii^	2.387 (2)	C624—H631	0.9900
O202—C214	1.310 (4)	C625—C626	1.503 (8)
N201—C202	1.336 (4)	C625—H632	0.9900
N201—C209	1.355 (4)	C625—H633	0.9900
C202—C203	1.406 (5)	C626—H634	0.9800
C202—C211	1.449 (5)	C626—H635	0.9800
C203—C204	1.362 (5)	C626—H636	0.9800
			
C123—Sn1—C119	171.11 (12)	C203—C202—C211	123.0 (3)
C123—Sn1—O102	91.76 (10)	C204—C203—C202	119.3 (3)
C119—Sn1—O102	96.61 (10)	C204—C203—H203	120.3
C123—Sn1—O101^i^	88.12 (9)	C202—C203—H203	120.3
C119—Sn1—O101^i^	89.37 (10)	C203—C204—C210	120.4 (3)
O102—Sn1—O101^i^	86.79 (7)	C203—C204—H204	119.8
C123—Sn1—N101	91.28 (10)	C210—C204—H204	119.8
C119—Sn1—N101	84.84 (10)	C206—C205—C210	118.8 (3)
O102—Sn1—N101	135.62 (8)	C206—C205—H205	120.6
O101^i^—Sn1—N101	137.56 (8)	C210—C205—H205	120.6
C123—Sn1—N112	94.63 (10)	C205—C206—C207	123.3 (3)
C119—Sn1—N112	91.05 (10)	C205—C206—H206	118.4
O102—Sn1—N112	69.89 (8)	C207—C206—H206	118.4
O101^i^—Sn1—N112	156.58 (8)	C208—C207—C206	120.6 (3)
N101—Sn1—N112	65.73 (8)	C208—C207—H207	119.7
C123—Sn1—O101	83.56 (9)	C206—C207—H207	119.7
C119—Sn1—O101	87.57 (10)	O201—C208—C207	125.0 (3)
O102—Sn1—O101	157.16 (7)	O201—C208—C209	118.5 (3)
O101^i^—Sn1—O101	70.76 (7)	C207—C208—C209	116.5 (3)
N101—Sn1—O101	67.02 (7)	N201—C209—C210	121.5 (3)
N112—Sn1—O101	132.65 (7)	N201—C209—C208	116.8 (3)
C108—O101—Sn1^i^	133.82 (18)	C210—C209—C208	121.7 (3)
C108—O101—Sn1	116.89 (18)	C204—C210—C205	123.9 (3)
Sn1^i^—O101—Sn1	109.24 (7)	C204—C210—C209	117.1 (3)
C114—O102—Sn1	122.12 (19)	C205—C210—C209	119.0 (3)
C102—N101—C109	119.9 (3)	N212—C211—C202	118.1 (3)
C102—N101—Sn1	120.6 (2)	N212—C211—H211	121.0
C109—N101—Sn1	119.51 (19)	C202—C211—H211	121.0
N101—C102—C103	122.6 (3)	C211—N212—C213	125.6 (3)
N101—C102—C111	115.2 (3)	C211—N212—Sn2	119.7 (2)
C103—C102—C111	122.1 (3)	C213—N212—Sn2	114.6 (2)
C104—C103—C102	118.4 (3)	C218—C213—N212	125.7 (3)
C104—C103—H103	120.8	C218—C213—C214	121.4 (3)
C102—C103—H103	120.8	N212—C213—C214	112.8 (3)
C103—C104—C110	120.8 (3)	O202—C214—C215	121.5 (3)
C103—C104—H104	119.6	O202—C214—C213	121.4 (3)
C110—C104—H104	119.6	C215—C214—C213	117.1 (3)
C106—C105—C110	118.8 (3)	C216—C215—C214	120.7 (4)
C106—C105—H105	120.6	C216—C215—H215	119.6
C110—C105—H105	120.6	C214—C215—H215	119.6
C105—C106—C107	122.4 (3)	C215—C216—C217	120.9 (3)
C105—C106—H106	118.8	C215—C216—H216	119.5
C107—C106—H106	118.8	C217—C216—H216	119.5
C108—C107—C106	121.5 (3)	C218—C217—C216	120.0 (3)
C108—C107—H107	119.3	C218—C217—H217	120.0
C106—C107—H107	119.3	C216—C217—H217	120.0
O101—C108—C107	124.7 (3)	C217—C218—C213	119.8 (4)
O101—C108—C109	118.9 (3)	C217—C218—H218	120.1
C107—C108—C109	116.3 (3)	C213—C218—H218	120.1
N101—C109—C110	121.0 (3)	C220—C219—Sn2	117.6 (2)
N101—C109—C108	117.5 (3)	C220—C219—H219	107.9
C110—C109—C108	121.4 (3)	Sn2—C219—H219	107.9
C105—C110—C104	123.2 (3)	C220—C219—H220	107.9
C105—C110—C109	119.4 (3)	Sn2—C219—H220	107.9
C104—C110—C109	117.2 (3)	H219—C219—H220	107.2
N112—C111—C102	118.0 (3)	C219—C220—C221	114.0 (3)
N112—C111—H111	121.0	C219—C220—H221	108.8
C102—C111—H111	121.0	C221—C220—H221	108.8
C111—N112—C113	125.9 (3)	C219—C220—H222	108.8
C111—N112—Sn1	120.3 (2)	C221—C220—H222	108.8
C113—N112—Sn1	113.80 (19)	H221—C220—H222	107.7
C118—C113—N112	126.1 (3)	C222—C221—C220	114.2 (5)
C118—C113—C114	121.4 (3)	C422—C221—C220	120.6 (11)
N112—C113—C114	112.4 (3)	C222—C221—H223	108.7
O102—C114—C115	121.1 (3)	C220—C221—H223	108.7
O102—C114—C113	121.7 (3)	C222—C221—H224	108.7
C115—C114—C113	117.2 (3)	C220—C221—H224	108.7
C116—C115—C114	120.6 (3)	H223—C221—H224	107.6
C116—C115—H115	119.7	C422—C221—H423	107.2
C114—C115—H115	119.7	C220—C221—H423	107.2
C115—C116—C117	121.1 (3)	C422—C221—H424	107.2
C115—C116—H116	119.5	C220—C221—H424	107.2
C117—C116—H116	119.5	H423—C221—H424	106.8
C118—C117—C116	119.4 (3)	C221—C222—H225	109.5
C118—C117—H117	120.3	C221—C222—H226	109.5
C116—C117—H117	120.3	H225—C222—H226	109.5
C117—C118—C113	120.2 (3)	C221—C222—H227	109.5
C117—C118—H118	119.9	H225—C222—H227	109.5
C113—C118—H118	119.9	H226—C222—H227	109.5
C120—C119—Sn1	117.0 (2)	C221—C422—H425	109.5
C120—C119—H119	108.0	C221—C422—H426	109.5
Sn1—C119—H119	108.0	H425—C422—H426	109.5
C120—C119—H120	108.0	C221—C422—H427	109.5
Sn1—C119—H120	108.0	H425—C422—H427	109.5
H119—C119—H120	107.3	H426—C422—H427	109.5
C119—C120—C121	112.3 (3)	C424—C223—Sn2	122.9 (6)
C119—C120—H121	109.1	C224—C223—Sn2	119.1 (6)
C121—C120—H121	109.1	C624—C223—Sn2	119.6 (7)
C119—C120—H122	109.1	C224—C223—H228	107.5
C121—C120—H122	109.1	Sn2—C223—H228	107.5
H121—C120—H122	107.9	C224—C223—H229	107.5
C122—C121—C120	113.8 (4)	Sn2—C223—H229	107.5
C122—C121—H123	108.8	H228—C223—H229	107.0
C120—C121—H123	108.8	C424—C223—H428	106.6
C122—C121—H124	108.8	Sn2—C223—H428	106.6
C120—C121—H124	108.8	C424—C223—H429	106.6
H123—C121—H124	107.7	Sn2—C223—H429	106.6
C121—C122—H125	109.5	H428—C223—H429	106.6
C121—C122—H126	109.5	C624—C223—H628	107.4
H125—C122—H126	109.5	Sn2—C223—H628	107.4
C121—C122—H127	109.5	C624—C223—H629	107.4
H125—C122—H127	109.5	Sn2—C223—H629	107.4
H126—C122—H127	109.5	H628—C223—H629	107.0
C124—C123—Sn1	116.1 (2)	C223—C224—C225	116.5 (7)
C124—C123—H128	108.3	C223—C224—H230	108.2
Sn1—C123—H128	108.3	C225—C224—H230	108.2
C124—C123—H129	108.3	C223—C224—H231	108.2
Sn1—C123—H129	108.3	C225—C224—H231	108.2
H128—C123—H129	107.4	H230—C224—H231	107.3
C125—C124—C123	111.9 (2)	C224—C225—C226	115.3 (7)
C125—C124—H130	109.2	C224—C225—H232	108.4
C123—C124—H130	109.2	C226—C225—H232	108.4
C125—C124—H131	109.2	C224—C225—H233	108.4
C123—C124—H131	109.2	C226—C225—H233	108.4
H130—C124—H131	107.9	H232—C225—H233	107.5
C126—C125—C124	113.5 (3)	C225—C226—H234	109.5
C126—C125—H132	108.9	C225—C226—H235	109.5
C124—C125—H132	108.9	H234—C226—H235	109.5
C126—C125—H133	108.9	C225—C226—H236	109.5
C124—C125—H133	108.9	H234—C226—H236	109.5
H132—C125—H133	107.7	H235—C226—H236	109.5
C125—C126—H134	109.5	C223—C424—C425	112.8 (9)
C125—C126—H135	109.5	C223—C424—H430	109.0
H134—C126—H135	109.5	C425—C424—H430	109.0
C125—C126—H136	109.5	C223—C424—H431	109.0
H134—C126—H136	109.5	C425—C424—H431	109.0
H135—C126—H136	109.5	H430—C424—H431	107.8
C223—Sn2—C219	171.72 (13)	C426—C425—C424	114.0 (7)
C223—Sn2—O202	94.31 (12)	C426—C425—H432	108.8
C219—Sn2—O202	90.04 (11)	C424—C425—H432	108.8
C223—Sn2—N201	91.86 (12)	C426—C425—H433	108.8
C219—Sn2—N201	89.92 (11)	C424—C425—H433	108.8
O202—Sn2—N201	135.74 (9)	H432—C425—H433	107.7
C223—Sn2—O201^ii^	84.08 (11)	C425—C426—H434	109.5
C219—Sn2—O201^ii^	88.98 (10)	C425—C426—H435	109.5
O202—Sn2—O201^ii^	88.74 (8)	H434—C426—H435	109.5
N201—Sn2—O201^ii^	135.51 (8)	C425—C426—H436	109.5
C223—Sn2—N212	91.52 (11)	H434—C426—H436	109.5
C219—Sn2—N212	96.61 (11)	H435—C426—H436	109.5
O202—Sn2—N212	69.71 (9)	C625—C624—C223	112.9 (15)
N201—Sn2—N212	66.35 (9)	C625—C624—H630	109.0
O201^ii^—Sn2—N212	157.66 (8)	C223—C624—H630	109.0
C223—Sn2—O201	88.93 (11)	C625—C624—H631	109.0
C219—Sn2—O201	84.30 (10)	C223—C624—H631	109.0
O202—Sn2—O201	156.48 (8)	H630—C624—H631	107.8
N201—Sn2—O201	67.24 (8)	C626—C625—C624	116.2 (9)
O201^ii^—Sn2—O201	68.40 (8)	C626—C625—H632	108.2
N212—Sn2—O201	133.57 (8)	C624—C625—H632	108.2
C208—O201—Sn2^ii^	131.56 (19)	C626—C625—H633	108.2
C208—O201—Sn2	116.34 (18)	C624—C625—H633	108.2
Sn2^ii^—O201—Sn2	111.60 (8)	H632—C625—H633	107.4
C214—O202—Sn2	120.6 (2)	C625—C626—H634	109.5
C202—N201—C209	119.9 (3)	C625—C626—H635	109.5
C202—N201—Sn2	120.4 (2)	H634—C626—H635	109.5
C209—N201—Sn2	119.5 (2)	C625—C626—H636	109.5
N201—C202—C203	121.6 (3)	H634—C626—H636	109.5
N201—C202—C211	115.4 (3)	H635—C626—H636	109.5
			
C109—N101—C102—C103	0.1 (4)	Sn2—N201—C202—C211	0.3 (4)
Sn1—N101—C102—C103	−179.5 (2)	N201—C202—C203—C204	3.5 (5)
C109—N101—C102—C111	−178.0 (3)	C211—C202—C203—C204	−175.8 (3)
Sn1—N101—C102—C111	2.4 (4)	C202—C203—C204—C210	0.1 (5)
N101—C102—C103—C104	−1.1 (5)	C210—C205—C206—C207	−3.1 (5)
C111—C102—C103—C104	176.9 (3)	C205—C206—C207—C208	0.6 (5)
C102—C103—C104—C110	1.6 (5)	Sn2^ii^—O201—C208—C207	4.5 (4)
C110—C105—C106—C107	1.2 (5)	Sn2—O201—C208—C207	−166.6 (2)
C105—C106—C107—C108	2.0 (5)	Sn2^ii^—O201—C208—C209	−174.74 (19)
Sn1^i^—O101—C108—C107	−8.3 (4)	Sn2—O201—C208—C209	14.2 (3)
Sn1—O101—C108—C107	174.6 (2)	C206—C207—C208—O201	−175.7 (3)
Sn1^i^—O101—C108—C109	172.68 (18)	C206—C207—C208—C209	3.5 (4)
Sn1—O101—C108—C109	−4.4 (3)	C202—N201—C209—C210	0.0 (4)
C106—C107—C108—O101	176.9 (3)	Sn2—N201—C209—C210	175.5 (2)
C106—C107—C108—C109	−4.0 (4)	C202—N201—C209—C208	−177.4 (3)
C102—N101—C109—C110	0.3 (4)	Sn2—N201—C209—C208	−1.9 (4)
Sn1—N101—C109—C110	179.9 (2)	O201—C208—C209—N201	−8.5 (4)
C102—N101—C109—C108	177.8 (3)	C207—C208—C209—N201	172.1 (3)
Sn1—N101—C109—C108	−2.6 (3)	O201—C208—C209—C210	174.1 (3)
O101—C108—C109—N101	4.7 (4)	C207—C208—C209—C210	−5.2 (4)
C107—C108—C109—N101	−174.4 (3)	C203—C204—C210—C205	174.3 (3)
O101—C108—C109—C110	−177.8 (3)	C203—C204—C210—C209	−3.4 (5)
C107—C108—C109—C110	3.1 (4)	C206—C205—C210—C204	−176.4 (3)
C106—C105—C110—C104	174.8 (3)	C206—C205—C210—C209	1.3 (5)
C106—C105—C110—C109	−2.0 (5)	N201—C209—C210—C204	3.5 (4)
C103—C104—C110—C105	−178.1 (3)	C208—C209—C210—C204	−179.2 (3)
C103—C104—C110—C109	−1.2 (5)	N201—C209—C210—C205	−174.4 (3)
N101—C109—C110—C105	177.3 (3)	C208—C209—C210—C205	2.9 (5)
C108—C109—C110—C105	−0.2 (5)	N201—C202—C211—N212	−2.1 (4)
N101—C109—C110—C104	0.2 (4)	C203—C202—C211—N212	177.2 (3)
C108—C109—C110—C104	−177.2 (3)	C202—C211—N212—C213	179.0 (3)
N101—C102—C111—N112	1.2 (4)	C202—C211—N212—Sn2	2.9 (4)
C103—C102—C111—N112	−176.9 (3)	C211—N212—C213—C218	−3.4 (5)
C102—C111—N112—C113	176.2 (3)	Sn2—N212—C213—C218	172.9 (3)
C102—C111—N112—Sn1	−4.3 (4)	C211—N212—C213—C214	178.5 (3)
C111—N112—C113—C118	−4.9 (5)	Sn2—N212—C213—C214	−5.2 (3)
Sn1—N112—C113—C118	175.5 (3)	Sn2—O202—C214—C215	−171.8 (2)
C111—N112—C113—C114	177.2 (3)	Sn2—O202—C214—C213	9.0 (4)
Sn1—N112—C113—C114	−2.3 (3)	C218—C213—C214—O202	−180.0 (3)
Sn1—O102—C114—C115	−175.9 (2)	N212—C213—C214—O202	−1.8 (4)
Sn1—O102—C114—C113	3.6 (4)	C218—C213—C214—C215	0.7 (5)
C118—C113—C114—O102	−178.5 (3)	N212—C213—C214—C215	178.9 (3)
N112—C113—C114—O102	−0.5 (4)	O202—C214—C215—C216	179.6 (3)
C118—C113—C114—C115	1.1 (4)	C213—C214—C215—C216	−1.1 (5)
N112—C113—C114—C115	179.1 (3)	C214—C215—C216—C217	1.2 (6)
O102—C114—C115—C116	179.0 (3)	C215—C216—C217—C218	−0.8 (5)
C113—C114—C115—C116	−0.6 (5)	C216—C217—C218—C213	0.4 (5)
C114—C115—C116—C117	0.5 (5)	N212—C213—C218—C217	−178.3 (3)
C115—C116—C117—C118	−0.9 (5)	C214—C213—C218—C217	−0.4 (5)
C116—C117—C118—C113	1.4 (5)	Sn2—C219—C220—C221	172.1 (3)
N112—C113—C118—C117	−179.2 (3)	C219—C220—C221—C222	63.0 (8)
C114—C113—C118—C117	−1.5 (5)	C219—C220—C221—C422	38.4 (13)
Sn1—C119—C120—C121	−174.7 (3)	Sn2—C223—C224—C225	168.1 (9)
C119—C120—C121—C122	−171.7 (4)	C223—C224—C225—C226	−97.6 (16)
Sn1—C123—C124—C125	178.1 (2)	Sn2—C223—C424—C425	176.8 (9)
C123—C124—C125—C126	179.6 (3)	C223—C424—C425—C426	−172.6 (15)
C209—N201—C202—C203	−3.5 (5)	Sn2—C223—C624—C625	−171.8 (12)
Sn2—N201—C202—C203	−179.1 (2)	C223—C624—C625—C626	129 (2)
C209—N201—C202—C211	175.8 (3)		

Symmetry codes: (i) −*x*+1, −*y*+1, −*z*; (ii) −*x*+1, −*y*, −*z*+1.

### Hydrogen-bond geometry (Å, º)

*Cg*2, *Cg*3, *Cg*4 and *Cg*5 are the centroids of the C205–C210, C213–C218, N101/C104/C109C110 and C105–C110 rings, respectively.

**Table:**

*D*—H···*A*	*D*—H	H···*A*	*D*···*A*	*D*—H···*A*
C107—H107···O102^i^	0.95	2.09	2.908 (4)	144
C207—H207···O202^ii^	0.95	2.18	2.972 (4)	140
C105—H105···*Cg*2	0.95	2.71	3.609 (3)	159
C116—H116···*Cg*3^iii^	0.95	2.94	3.704 (3)	139
C125—H132···*Cg*2^iv^	0.99	2.77	3.602 (3)	142
C204—H204···*Cg*5^v^	0.95	2.77	3.700 (4)	165
C222—H226···*Cg*4^ii^	0.98	2.69	3.613 (14)	157
C422—H426···*Cg*5^ii^	0.98	2.94	3.77 (3)	142

Symmetry codes: (i) −*x*+1, −*y*+1, −*z*; (ii) −*x*+1, −*y*, −*z*+1; (iii) *x*−1, *y*+1, *z*−1; (iv) *x*, *y*, *z*−1; (v) −*x*+1, −*y*+1, −*z*+1.
